# Synergistic Effect of Static Magnetic Fields and 3D-Printed Iron-Oxide-Nanoparticle-Containing Calcium Silicate/Poly-ε-Caprolactone Scaffolds for Bone Tissue Engineering

**DOI:** 10.3390/cells11243967

**Published:** 2022-12-08

**Authors:** Chuan-Yi Kao, Tsung-Li Lin, Yen-Hong Lin, Alvin Kai-Xing Lee, Sing Yee Ng, Tsui-Hsien Huang, Tuan-Ti Hsu

**Affiliations:** 1 School of Medicine, Chung Shan Medical University, Taichung 406040, Taiwan; 2 Department of Psychiatry, Chung Shan Medical University Hospital, Taichung 40201, Taiwan; 3 Department of Sports Medicine, College of Health Care, China Medical University, Taichung 406040, Taiwan; 4 Graduate Institute of Biomedical Sciences, China Medical University, Taichung 406040, Taiwan; 5 Department of Orthopedics, China Medical University Hospital, Taichung 40447, Taiwan; 6 x-Dimension Center for Medical Research and Translation, China Medical University Hospital, Taichung 404332, Taiwan; 7 Department of Education, China Medical University Hospital, Taichung 404332, Taiwan; 8 Department of Internal Medicine, Hospital Kuala Lumpur, Kuala Lumpur 50586, Malaysia; 9 School of Dentistry, Chung Shan Medical University, Taichung 406040, Taiwan; 10 Department of Stomatology, Chung Shan Medical University Hospital, Taichung 40201, Taiwan

**Keywords:** static magnetic fields, iron oxide, calcium silicate, bone regeneration, 3D scaffolds

## Abstract

In scaffold-regulated bone regeneration, most three-dimensional (3D)-printed scaffolds do not provide physical stimulation to stem cells. In this study, a magnetic scaffold was fabricated using fused deposition modeling with calcium silicate (CS), iron oxide nanoparticles (Fe_3_O_4_), and poly-ε-caprolactone (PCL) as the matrix for internal magnetic sources. A static magnetic field was used as an external magnetic source. It was observed that 5% Fe_3_O_4_ provided a favorable combination of compressive strength (9.6 ± 0.9 MPa) and degradation rate (21.6 ± 1.9% for four weeks). Furthermore, the Fe_3_O_4_-containing scaffold increased in vitro bioactivity and Wharton’s jelly mesenchymal stem cells’ (WJMSCs) adhesion. Moreover, it was shown that the Fe_3_O_4_-containing scaffold enhanced WJMSCs’ proliferation, alkaline phosphatase activity, and the osteogenic-related proteins of the scaffold. Under the synergistic effect of the static magnetic field, the CS scaffold containing Fe_3_O_4_ can not only enhance cell activity but also stimulate the simultaneous secretion of collagen I and osteocalcin. Overall, our results demonstrated that Fe_3_O_4_-containing CS/PCL scaffolds could be fabricated three dimensionally and combined with a static magnetic field to affect cell behaviors, potentially increasing the likelihood of clinical applications for bone tissue engineering.

## 1. Introduction

Large bone defects present a major clinical challenge and hurdle for surgeons due to the limited intrinsic regenerative capabilities of bones [1]. Scaffolds play a role in bone regeneration owing to their ability to act as a supportive bridge between a suitable microenvironment and a regenerative niche for osteogenesis [2]. Traditionally, ideal scaffolds for bone regeneration should have high bioactivity, biodegradability, and mechanical properties sufficient for structural support [3]. These properties have been extensively studied over the last few decades, and traditional studies on bone regeneration scaffolds frequently emphasize the importance of pore size, porosity, pore structure, and pore interconnectivity, which are all critical factors for efficient bone tissue regeneration [4,5]. The development and emergence of three-dimensional (3D) printing and fabrication technologies have enabled the fabrication of scaffolds with specific designs and parameters [6,7]. Calcium silicate (CS)-based bioceramics have become an area of interest for bone tissue engineering because of their excellent bioactivity, biocompatibility, and high mechanical properties [8]. In addition, CS scaffolds have been shown to release silicate ions (Si) in a stable and gradual manner. Si is an important trace element in the human body, and the presence of Si ions stimulates the osteogenic differentiation of stem cells in the absence of osteogenic-related factors [9]. Furthermore, Si ions have also been shown to induce the proliferation and differentiation of human umbilical vein endothelial stem cells by inducing the secretion of vascular endothelial growth factors [9,10]. However, we have now reached a stalemate in bone tissue regeneration, necessitating additional research to bring CS scaffolds closer to clinical applications [11].

Recent studies have demonstrated that mechanical force stimulation is an effective promoter and inducer of bone formation and regeneration [12,13,14]. This concept stemmed from clinical observations that patients who were less active or paralyzed had poor bone regeneration capabilities, as well as muscle atrophy and osteoporosis, as a result of disuse-induced bone loss with a lack of mechanical stimulation [15,16]. Therefore, various studies have attempted to study the relationship between mechanical stimulation and bone tissue regeneration, with the majority focusing on determining the optimal extrinsic stimulation for efficient bone growth and regeneration. Extrinsic mechanical stimulations, such as vibration, ultrasound, and electrical stimulation, have been shown to promote bone regeneration and enhance fracture healing [17,18,19,20]. Because of the development of magnetic nanoparticles (MNPs), magnetic stimulation has been reported to have the best potential for effective bone regenerative capabilities [21,22,23]. MNPs have been introduced into various matrices and platforms in order to induce stimuli-responsive regenerative properties for osteogenesis [24,25,26]. Interestingly, MNPs incorporated into calcium phosphate or polycaprolactone (PCL) polymer scaffolds have been shown to increase protein adsorption, resulting in increased cellular attachment and subsequent cellular proliferation [27,28,29]. A PCL polymer used to prepare bone scaffolds with ceramic powder, such as apatite and calcium phosphate, not only had a favorable mechanical performance but also enhanced bioactivity [30,31]. Furthermore, hydroxyapatite and chitosan/collagen scaffolds incorporated with MNPs have also been shown to have increased mechanical and osteogenic properties. According to the findings of these studies, MNPs can provide paramagnetic effects, which can then induce osteoblast differentiation and proliferation in a manner similar to the mechano-related pathways of extrinsic mechanical stimulation. MNPs have shown potential for bone tissue regeneration, and further studies are warranted to confirm their clinical application in bone regeneration [32].

To the best of our knowledge, there are currently no studies combining CS and MNPs for bone tissue regeneration. In this study, we fabricated porous CS scaffolds with Fe_3_O_4_ nanoparticles using 3D printing and evaluated their capability to regenerate bone tissue. We hypothesized that the paramagnetic effects of Fe_3_O_4_ would further enhance the regenerative capabilities of the CS scaffolds. In this study, we fabricated nano-Fe scaffolds of varying concentrations and determined their basic physicochemical properties and osteogenic capabilities by evaluating osteogenic-related markers. Our results showed that the incorporation of Fe_3_O_4_ into CS scaffolds further enhanced the mechanical strength and increased the secretion of osteogenic-related markers, such as alkaline phosphatase (ALP), bone sialoprotein (BSP), collagen I (COLI), and osteocalcin (OC). Taken together, we hope that this study will serve as a platform for future Fe_3_O_4_-related studies, bringing bone tissue regeneration a step closer to clinical applications.

## 2. Materials and Methods

### 2.1. Fabrication of Scaffolds

The protocols for the CS scaffold fabrication were adopted from our previous studies [33]. To obtain pure CS powder, CaO (99.9%, Sigma-Aldrich, St. Louis, MO, USA), SiO_2_ (1 µm, 99.99%, Sigma-Aldrich), and Al_2_O_3_ (99.5%, Sigma-Aldrich) were added and mixed in proportions of 70%, 25%, and 5%, respectively, followed by sintering in a high-temperature furnace at 1400 °C for 2 h. Subsequently, varying amounts of Fe_3_O_4_ nanoparticles (100 nm, 97%, Sigma-Aldrich) were added to the CS powder (0, 2.5, and 5 wt. %) and ball-milled at 300 rpm for 12 h. The ball-milled product was then dried and mixed in a 1:1 ratio with poly-ε-caprolactone (PCL; Mw 43,000; Polysciences, Warrington, PA, USA). First, PCL was melted at 180 °C, then alcohol was added and uniformly stirred before being placed in an oven for 12 h of dehydration. The CS-PCL-Fe pastes were stored in a remodified storage until further use. Prior to printing, the pastes were placed into the printing cartridge with a 20G nozzle, heated to 80 °C, and printed at 2 mm/s and 240 kPa. The various groups were named according to the concentration of Fe_3_O_4_ used, namely Fe0, Fe2.5, and Fe5.

### 2.2. Physicochemical Properties of the Scaffolds

The scaffolds for physicochemical properties analysis were designed to be a height of 10 mm and were stacked to each other at a 0°–90° orientation. The first two layers of the scaffold were both filled by seven parallel struts with a diameter of 500 μm and separated by 500 µm. Subsequent third and fourth layers were filled by six parallel struts which were located above the gaps of first and second layers, respectively. The compressive strength of the printed scaffolds was measured using a universal material-testing machine (EZ-TEST, Shimadzu, Kyoto, Japan) at a compression speed of 1 mm/min. The scaffolds were compressed until fracturing, and the final compressive strength was recorded. Six samples per group were tested, and the average values are presented. Furthermore, the surface morphology was observed using an optical microscope, and the microstructures were analyzed using field-emission scanning electron microscopy (FE-SEM; JSM-7800F, JEOL, Tokyo, Japan).

### 2.3. In Vitro Immersion Experiment

The scaffolds were placed in 15 mL of simulated body fluid (SBF) and placed in a water bath at 37 °C for different durations. The weights of the scaffolds were recorded before and after immersion, and the differences in weight were recorded as the percentage weight loss. Six samples per group were tested, and the average values are presented. The microstructures of the scaffolds were observed using FE-SEM.

### 2.4. Magnetic Stimulation

Each group received 20 min of magnetic stimulation daily. The control group, without Fe_3_O_4_ (Fe0), was also stimulated and cultured under similar conditions.

### 2.5. Cell Proliferation and Morphology

In the in vitro study, we used a scaffold with a diameter of 6 mm and a height of 2 mm which followed the same structural design. The cell proliferation, osteogenic differentiation, and cytokine secretion were tested using Wharton’s jelly mesenchymal stem cells (WJMSCs) purchased from the Bioresource Conservation and Research Center (BCRC; Hsinchu, Taiwan). Cells were cultured in Dulbecco’s modified Eagle’s medium (DMEM) containing 10% fetal bovine serum (FBS) and 1% antibiotics in an incubator with 5% CO_2_ at 37 °C, with medium change every two days. Cellular proliferation was determined after 1 and 7 d of culture using PrestoBlue^®^ (Invitrogen, Grand Island, NY, USA). Briefly, the reagent was mixed at 1:9 PrestroBlue to DMEM ratio, and the scaffolds were left to react for 1 h. After being removed from the reagent, the scaffolds were rinsed with phosphate-buffered saline (PBS) and then fixed with 4% paraformaldehyde. The absorbance of the reagent was measured at 570 nm using a Tecan Infinite 200 PRO microplate reader (Tecan, Männedorf, Switzerland) with a reference wavelength of 600 nm. Six samples per group were tested, and the average values are presented.

For morphological analysis, scaffolds were fixed with 4% paraformaldehyde for 20 min and permeabilized with 1% Triton X-100. The cytoskeleton was stained with a fluorescent dye (Alexa Fluor 488, Invitrogen) conjugated with phalloidin, and the nucleus was stained using DAPI (4′,6-diamidino-2-phenylindole, dilactate). Images were captured and analyzed using a Leica TCS SP8 X white light laser confocal microscope (Leica Microsystems GmbH, Wetzlar, Germany).

### 2.6. Osteogenic Differentiation

Alkaline phosphatase activity (ALP), bone sialoprotein (BSP), collagen I (COLI), and osteocalcin (OC) expression were measured at different time points to evaluate the osteogenic capabilities of the scaffolds. For ALP evaluation, cells were lysed in 100 μL of 1% NP40 buffer and evaluated using a pNPP alkaline phosphatase assay kit (BioAssay Systems, Biocore, New South Wales, Australia). The total protein content was measured using a BCA Protein Assay Kit (Thermo Scientific, Waltham, MA, USA). ALP activity was calculated as the difference in absorbance divided by the total protein content. Protein levels were determined using an enzyme-linked immunosorbent assay (ELISA) according to the manufacturer’s instructions for BSP, COLI, and OC evaluations.

### 2.7. Statistical Analysis

In each experiment, a one-way statistical analysis of variance (ANOVA) was used to analyze the significance of the differences between the different experimental groups. Significant deviation of each sample was determined using Scheffe’s multiple comparison test. A *p*-value < 0.05 was considered statistically significant, as indicated by “*” or “#” in the different group comparisons.

## 3. Results and Discussion

### 3.1. Characterization of the Physicochemical Properties of the Scaffolds 

**Figure 1:**
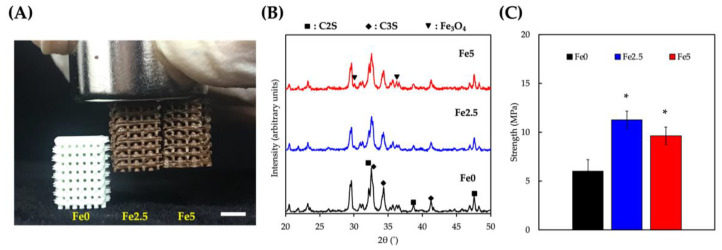
(**A**) Photo image of the side view of the 3D-printed scaffolds exhibiting their color change when adding MNP, and attracted using hand-held magnet. Scale bar is 3 mm. (**B**) XRD analysis and (**C**) compressive strength and modulus of Fe0, Fe2.5, and Fe5, respectively, * *p* < 0.05.

In this study, we used direct ink writing (DIW) to fabricate porous CS/PCL scaffolds with various concentrations of iron oxide (Fe_3_O_4_) nanoparticles. As shown in Figure 1A, the addition of Fe_3_O_4_ gave the scaffolds a brownish appearance depending on the concentration of Fe_3_O_4_ added. Furthermore, the addition of Fe_3_O_4_ did not affect the printing quality of the scaffolds, as indicated by the uniform porosity of the scaffolds. It was earlier reported that 400–600 μm pores were optimal for bone tissue regeneration; thus, the scaffolds used in this study had pore sizes of approximately 500 ± 10 µm [34]. As shown in Figure 1A, the scaffolds with Fe_3_O_4_ MNPs exhibited magnetic properties and could be attracted using hand-held magnets. Additive manufacturing is a layer-by-layer fabrication technique that has become popular in the last decade for producing 3D porous biodegradable magnetic scaffolds with interconnected pores for bone substitutes. Compared with traditional manufacturing methods, additive manufacturing has the potential to fabricate porous scaffolds with complex geometries and flexibility [35]. However, due to the magnetic capabilities of MNPs, it was discovered during the fabrication phase that traditional magnetic stirring could not be used in such studies involving MNPs. Therefore, we added high-molecular-weight PCL to CS to increase the viscosity, allowing the MNPs to be distributed uniformly and eliminating external environmental influences [36]. It can be observed from the appearance of the printed scaffolds that the MNPs were homogeneously distributed throughout the scaffold [27].

Since both CS and Fe_3_O_4_ are crystalline compounds, X-ray diffraction (XRD) was used for the phase identification of the scaffolds. As shown in Figure 1B, the characteristic peaks at 32.5°, 38.7°, and 47.6° correspond to C2S, whereas the characteristic peaks at 32.7°, 34.32°, and 41.2° correspond to C3S [37]. The characteristic peaks at 30.1° and 36.2° correspond to Fe_3_O_4_ [38]. Furthermore, the peak intensities of Fe_3_O_4_ increased with increasing MNP content, indicating that different concentrations of Fe_3_O_4_ could be added to CS/PCL scaffolds. Furthermore, no other peaks were observed in the scaffolds, indicating that Fe_3_O_4_ modification did not alter or influence the original structural properties of CS/PCL. The compressive strength of the scaffold samples was evaluated by compression testing, and the results are shown in Figure 1C. The Fe0 scaffolds had a compressive strength of approximately 5.2 ± 0.2 MPa. In Fe2.5, the compressive strength increased significantly to 11.5 ± 0.6 MPa, which was similar to the compressive strength of native cancellous bone [39]. However, the compressive strength of Fe5 is 9.9 ± 0.4 MPa, which is not significantly different from Fe2.5 (*p* > 0.05), but still significantly higher than that of the Fe0 scaffold (*p* < 0.05). The results indicate that adding MNPs significantly improved the compressive performance of the scaffolds because of their effective resistance to polymer chain deformation under external forces as rigid particles [40]. In general, nanoparticles, as nanofillers, can improve the mechanical properties of polymer matrices owing to their nano-enhancing or rigidity-enhancing effects [41]. Fe_3_O_4_ MNPs are known to have a high level of stiffness and mechanical strength, which can be used to improve the mechanical properties of scaffolds owing to their stiffness-enhancing effects. In addition, the MNPs were homogenously distributed throughout the scaffolds, further increasing their weight-bearing and stress distribution effects [42]. This can be characteristic of the reinforced interactions between uniformly dispersed inflexible Fe_3_O_4_ particles in the CS/PCL composites which reduced the stress concentration and improved the ultimate stress [43]. From our results, it is worth noting that this effect could only be achieved when the nanoparticles were homogenously dispersed in the matrix, and that an excessive addition of nanoparticles can cause agglomeration, thus reducing the mechanical strength of the scaffolds.

### 3.2. Immersed Behaviors

**Figure 2:**
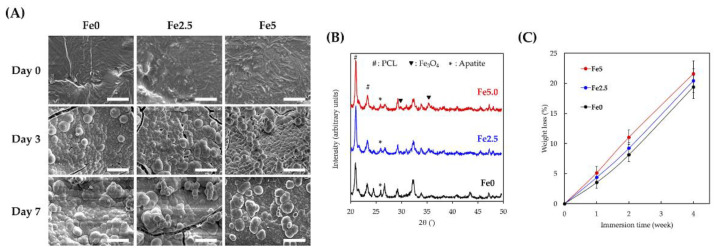
(**A**) Surface morphology and (**B**) XRD changes in the various scaffolds of immersion in SBF for different time points. Scale bar is 3 µm. (**C**) Weight loss of the Fe0, Fe2.5, and Fe5 during 4 weeks of immersion.

Figure 2A shows the surface morphology of the porous scaffolds after immersion in SBF for seven days. Prior to immersion (day 0), the surfaces of the scaffolds were relatively flat with slight elevations because of the native appearance of PCL after cooling. The entrapment of CS and MNPs by macromolecules resulted in numerous notches on the surface, which is consistent with our previous studies. After three days of immersion, the surfaces of the scaffolds were covered with a layer of precipitated hydroxyapatite aggregates several micrometers in size. Furthermore, the precipitated aggregates grew in size as the immersion time increased. However, unlike the typical “grape-like” morphology of hydroxyapatite aggregates on the surfaces of Fe0, the aggregates on the Fe2.5 and Fe5 surfaces were less dense and not closely packed [44]. In addition, the XRD profiles further verified the appearance of the HA peak at 25.9° in all the scaffolds which demonstrated that the CS-based scaffolds had high bioactivity [45]. Interestingly, the density of the precipitated products decreased as MNP concentration increased. Regardless, after immersion for three days, the surfaces of all groups were covered with a layer of precipitated hydroxyapatite, indicating that the scaffolds still had good bioactivity, even with the addition of MNPs. Figure 2C shows that the degradation rates remained constant and stable over the four weeks of immersion, and the final weight losses of Fe0, Fe2.5, and Fe5 were 19.4 ± 2.1%, 20.5 ± 2.0%, and 21.6 ± 1.9%, respectively. These results indicate that we could control and regulate in vitro degradation rates by altering and adjusting the MNP concentrations in porous scaffolds [46]. The biocompatibility of biodegradable implants is directly affected by their degradation properties (degradation rate and degradation products), which mainly depend on the material composition and structural characteristics [47]. The presence of MNPs in the scaffold was hypothesized to cause structural laxity among the CS, resulting in the increased degradation rate of the MNP scaffolds.

### 3.3. Cell Proliferation of Wharton’s Jelly Mesenchymal Stem Cells (WJMSCs) Cultured on Scaffolds

**Figure 3:**
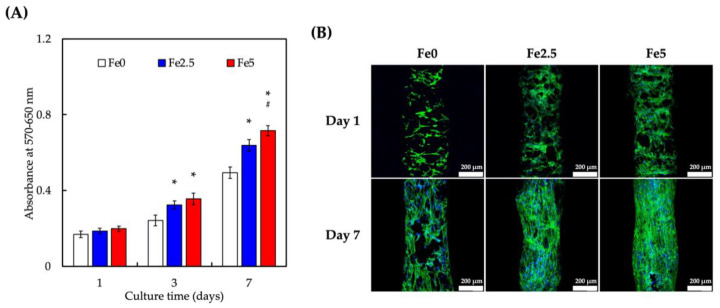
(**A**) The proliferation of WJMSCs cultured on Fe0, Fe2.5, and Fe5 for 1, 3, and 7 days. “*” indicates a significant difference (* *p* < 0.05) when compared to Fe0; “#” indicates a significant difference (# *p* < 0.05) when compared to Fe2.5. (**B**) The F-actin filaments (green) staining of WJMSCs cultured on scaffolds at different time points to observe cell morphological changes. The scale bar is 200 µm.

To examine the morphology and viability of WJMSCs, the scaffolds were tested for cytocompatibility in vitro using a PrestoBlue assay (Figure 3). A time-dependent increase in cellular proliferation was observed in all scaffolds. However, cellular proliferation was significantly higher on Fe2.5 and Fe5 on days 3 and 7 of culture, respectively (Figure 3A). In addition, on day 7 of culture, Fe5 had a significantly higher cellular proliferation than that of Fe2.5 and Fe0. The cellular morphology was observed using immunofluorescence staining, as shown in Figure 3B. Cells on all three scaffolds adhered well to the scaffold surfaces after day 1 of culture, as seen from their elongated and flattened morphology [48]. However, on day 7 of culture, cells on Fe2.5 and Fe5 showed an enhanced cellular attachment compared to those on Fe0, as seen from their morphologies. These results confirmed that the incorporation of MNPs into the scaffolds significantly improved the cell viability, adhesion, and spreading [49]. The biocompatibility of Fe_3_O_4_-containing scaffolds may be due to its surface energy and intrinsic magnetic properties, which have been shown to promote cellular adhesion and attachment. Our findings on MNPs and cellular responses were consistent with previous findings that magnetic hydroxyapatite scaffolds could improve the viability and proliferation of MG63 and MC3T3-E1 cells [50].

### 3.4. Effect of Ionic Scaffold Products on WJMSCs’ Differentiation

**Figure 4:**
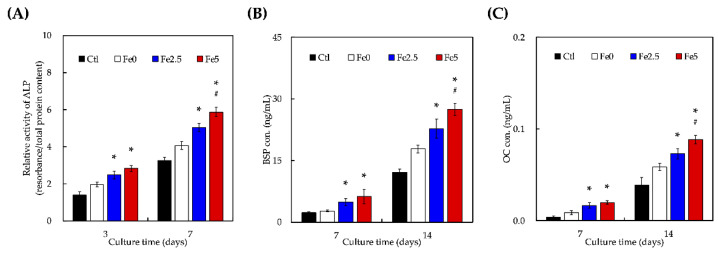
Osteogenic-related differentiation marker of (**A**) ALP, (**B**) BSP, and (**C**) OC expression of WJMSCs cultured on different Fe0, Fe2.5, and Fe5 at different time points. “*” indicates a significant difference (* *p* < 0.05) when compared to Fe0; “#” indicates a significant difference (# *p* < 0.05) when compared to Fe2.5.

The ALP activity was used as an indicator of the early stages of osteogenic differentiation, as shown in Figure 4A. As expected, the cells grown on all the scaffolds showed a time-dependent increase in their ALP activity. Cells grown on culture plates without scaffolds were used as controls. There were no significant differences in the ALP activity between the Fe0 and Ctl groups after day 3 of culture. However, after days 3 and 7 of culture, the ALP activity on the Fe2.5 and Fe5 scaffolds was significantly higher than that on the Fe0 scaffolds (*p* < 0.05). Notably, after day 7 of culture, the ALP activity of Fe5 was approximately 1.2-times higher than that of the Fe2.5 scaffolds (*p* < 0.05). Similar trends were noted for BSP and OC, which are both known markers of late-stage bone differentiation. These results indicate that the magnetic microenvironment provided by Fe_3_O_4_ nanoparticles in Fe_3_O_4_ scaffolds promoted cell proliferation and differentiation in a time- and dose-dependent manner [21]. Moreover, the stem cells cultured on the scaffold experienced increased osteogenic differentiation, ALP secretion, and calcium deposition compared to those seeded on scaffolds without Fe_3_O_4_, indicating that the osteogenic differentiation of stem cells was significantly regulated by the incorporation of Fe_3_O_4_ into ceramics [21]. Xia et al. demonstrated that Fe_3_O_4_-containing bioceramic scaffolds stimulated the osteogenic differentiation of stem cells and significantly upregulated the gene expression of WNT1, RUNX2, ALP, COL1, and OCN [51]. Moreover, β-catenin protein expression increased, which indicated that Fe_3_O_4_-containing bioceramic scaffolds activated Wnt/β-catenin signaling and downstream target genes. This result has been reported by other researchers using other biomaterials and cell types. Recent reports have also indicated that static magnetic stimulation can induce viability and osteogenesis both in vitro and in vivo [52]. Based on our results, we hypothesized that magnetic stimulation disrupted the arrangement of cellular membranes, influencing cellular adhesion and attachment and, as a result, increasing the secretion of osteogenesis-related markers. Magnetic stimulation has also been reported to regulate calcium regulation and secretion, thereby activating several downstream intercellular signaling pathways for osteogenesis [53].

### 3.5. Static Magnetic Fields Enhanced Cell Proliferation and Differentiation on FeCS Scaffold

**Figure 5:**
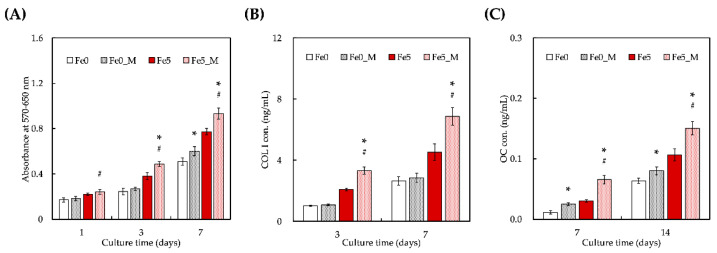
Evaluation of (**A**) cellular proliferation, (**B**) COLI, and (**C**) OC secretion from WJMSCs for osteogenic capability evaluation of the scaffolds after application of a MF. “*” indicates a significant difference (* *p* < 0.05) when compared to Fe0; “#” indicates a significant difference (# *p* < 0.05) when compared to Fe5.

We further evaluated the cellular proliferation and Col I and OC secretion cultured on the Fe-containing scaffolds with a static magnetic field (MF). The viability of WJMSCs on Fe0 and Fe5 with and without the application of a MF is shown in Figure 5A. After one day of culture, there was no significant difference in the cell viability between Fe0, Fe0_M, and Fe5. Notably, the cellular proliferation was significantly higher on Fe5_M (*p* < 0.05) on day 1 than on Fe5 without a MF. After day 3, the groups with an applied MF (Fe0_M and Fe5_M) showed significant differences from Fe0. On the other hand, cells cultured on Fe5_M exhibited more COL1 secretion (Figure 5B), which is the main organic component of the bone extracellular matrix, than the cells cultured on other scaffolds after days 3 and 7 of culture. Fe5_M also differed significantly from Fe0 and Fe5. In the later stage of osteogenesis, after days 7 and 14 of seeding, more abundant OC was distributed throughout Fe5_M compared with Fe5, and their trends were similar for both ALP and COLI. These results indicate that MNPs combined with a MF can promote osteogenic differentiation [54]. In vitro biocompatibility studies showed that the incorporation of MNPs was beneficial for the viability, proliferation, and differentiation of WJMSCs. Recent reports indicate that static magnetic fields not only enhance cell viability but also stimulate bone formation in vitro and in vivo [55,56]. From the above results, it can be reasonably inferred that MNPs can be regarded as a single magnetic domain that provides an intrinsic nanoscale magnetic field [57]. Thus, a microenvironment is constructed on a scaffold consisting of a large number of nanoscale magnetic fields. As a result, increasing the MNP content may improve overall stimulation of cell adhesion, proliferation, and differentiation [58]. One possible mechanism is that magnetic-stimulation-induced membrane phospholipid rearrangement activates further cell adhesion molecules, thereby enhancing cell adhesion. In addition, magnetic fields can modulate calcium ion levels, activate various signaling pathways, and improve the expression of osteogenesis-related growth factors, which are beneficial for enhancing and maintaining cell viability and inducing cell proliferation and differentiation. The interaction mechanism of MNPs with cell viability, proliferation, and differentiation requires further investigation.

## 4. Conclusions

In this study, a 3D-printed Fe_3_O_4_-containing CS/PCL scaffold with a biodegradable porous framework was fabricated using DIW. The porous scaffold not only exhibited cancellous bone-like mechanical properties but its mechanical properties could be adjusted by the addition of Fe_3_O_4_. In addition, the degradation rate of porous scaffolds can be tuned by varying the Fe_3_O_4_ concentration. The scaffolds were covered in a thick layer of precipitated hydroxyapatite, indicating that even with the addition of Fe_3_O_4_, the scaffolds retained good bioactivity. Cell viability assays showed the nontoxic properties of Fe_3_O_4_ at most concentrations, confirming its possible systemic application in medical applications. Most importantly, magnetized superparamagnetic scaffolds generate mechanical forces that drive WJMSCs’ growth toward an osteogenic lineage in scaffolds. Magnetic stimulation interferes with the arrangement of cellular membranes, leading to an increased secretion of osteogenic-related markers. We believe that this 3D print design for an Fe_3_O_4_-containing CS/PCL matrix could be used to interweave different biomaterials into a scaffold that combines static magnetic fields which play a vital role in bone tissue regeneration, improving the potential for use of these materials in bone tissue engineering and bringing it a step closer to clinical applications.

## Data Availability

Data are available in a publicly accessible repository.
